# Green tea drinking and subsequent risk of breast cancer in a population to based cohort of Japanese women

**DOI:** 10.1186/bcr2756

**Published:** 2010-10-28

**Authors:** Motoki Iwasaki, Manami Inoue, Shizuka Sasazuki, Norie Sawada, Taiki Yamaji, Taichi Shimazu, Walter C Willett, Shoichiro Tsugane

**Affiliations:** 1 Epidemiology and Prevention Division, Research Center for Cancer Prevention and Screening, National Cancer Center, 5 - 1 - 1 Tsukiji, Chuo - ku, Tokyo, 104 - 0045, Japan; 2 Departments of Nutrition and Epidemiology, Harvard School of Public Health, and Channing Laboratory, Department of Medicine, Brigham and Women's Hospital and Harvard Medical School, 665 Huntington Avenue, Boston, MA 02115, USA

## Abstract

**Introduction:**

Although many *in vitro *and animal studies have demonstrated a protective effect of green tea against breast cancer, findings from epidemiological studies have been inconsistent, and whether high green tea intake reduces the risk of breast cancer remains unclear.

**Methods:**

In this Japan Public Health Center-based Prospective Study, 581 cases of breast cancer were newly diagnosed in 53,793 women during 13.6 years' follow-up from the baseline survey in 1990 to 1994. After the five-year follow-up survey in 1995 to 1998, 350 cases were newly diagnosed in 43,639 women during 9.5 years' follow-up. The baseline questionnaire assessed the frequency of total green tea drinking while the five-year follow-up questionnaire assessed that of two types of green tea, *Sencha *and *Bancha/Genmaicha*, separately.

**Results:**

Compared with women who drank less than one cup of green tea per week, the adjusted hazard ratio (HR) for women who drank five or more cups per day was 1.12 (95% confidence interval (CI) 0.81 to 1.56; *P *for trend = 0.60) in the baseline data. Similarly, compared with women who drank less than one cup of *Sencha *or *Bancha/Genmaicha *per week, adjusted HRs for women who drank 10 or more cups per day were 1.02 (95% CI 0.55 to 1.89; *P *for trend = 0.48) for *Sencha *and 0.86 (0.34 to 2.17; *P *for trend = 0.66) for *Bancha/Genmaicha*. No inverse association was found regardless of hormone receptor-defined subtype or menopausal status.

**Conclusions:**

In this population-based prospective cohort study in Japan we found no association between green tea drinking and risk of breast cancer.

## Introduction

Green tea is regularly consumed in Japan and China as a traditional habit and cultural characteristic. Although produced from the same plant, *Camellia sinensis*, differences in the manufacturing process mean that green tea has a higher catechin content than black tea [1-3], which might contribute to its beneficial effects on cancer as well as cardiovascular diseases, and other conditions [3,4]. Specifically, (-)-epigallocatechin-3-gallate (EGCG), the most abundant and biologically active catechin in green tea, might play an important role in cancer prevention [1-3,5,6]. Because breast cancer risk is substantially lower in Asian than Western countries [7], a contribution of high green tea intake to low breast cancer risk has been hypothesized. This hypothesis has been supported by *in vitro *and animal studies, which have demonstrated various protective effects of green tea and tea polyphenols acting via strong antioxidant activity, inhibition of cell proliferation and angiogenesis, induction of apoptosis, and antiestrogenic properties [1-3,5,6,8].

In contrast to *in vitro *and animal studies, few epidemiological studies have examined the association between green tea intake and risk of breast cancer, and their findings have been inconsistent [9-16]. An inverse association was found in three case-control studies among Asian-American and Chinese populations [9-11], whereas no association was observed in two cohort studies in Japan [12,13] or one nested case-control study in Singapore [14]. This inconsistency might be in part explained by differences in study design and exposure variation. In the three case-control studies which reported an inverse association, the reference groups were non-green tea drinkers, which included approximately 36 to 68% of control subjects [9-11], whereas in the two Japanese cohort studies the reference groups were women who drank less than one cup per day or one or fewer cups per day [12,13]. Inclusion of green tea drinkers in the reference group might attenuate the difference in risk between the reference and the higher drinking groups. Moreover, the highest consumption group in the two Japanese cohort studies were women who drank five or more cups per day, which included a relatively large portion of subjects (approximately 26 to 43%) [12,13]. Several studies have suggested a possible protective effect of very large amounts of green tea intake: one case-control study in Japan showed a decreased risk of gastric cancer among those who drank 10 or more cups per day [17], for example, while a cohort study in Japan observed a decreased risk of cancer in all sites among those who drank 10 or more cups per day [18]. However, a better understanding of the role of green tea in the etiology of breast cancer would be obtained from studies which included non- to heavy green tea drinkers. In addition, given that *in vitro *studies have suggested the inhibition of aromatase by green tea extracts [8] and of estrogen binding with its receptor by EGCG [6], the effect of green tea intake on the risk of breast cancer may differ according to hormone receptor to defined subtype. Nevertheless, no study has evaluated these associations.

To address these issues, we conducted a large-scale population-based prospective cohort study in Japan on the association between green tea intake and the risk of breast cancer.

## Materials and methods

### Study population

The Japan Public Health Center-based Prospective Study (JPHC Study), which began in 1990 for Cohort I and in 1993 for Cohort II, included 140,420 inhabitants (68,722 men and 71,698 women) in the municipalities supervised by 11 public health centers (PHC). Details of the study design have been described elsewhere [19]. Study participants were informed about the objectives and methods of the study in written form and those who responded to the survey questionnaire were regarded as consenting to participate in the study. In addition, participants were also notified in writing that they could withdraw from the study. Given no relevant ethical guideline and committee at the time of the survey, the institutional review board of the National Cancer Center, Tokyo, Japan considered return of the questionnaire to be classified as informed consent and approved the study protocol.

The study population comprised registered Japanese inhabitants living in each PHC area, aged 40 to 59 years in Cohort I and 40 to 69 years in Cohort II. In the present analysis, one PHC area was excluded because data on cancer incidence were not available. Thus, after exclusion of ineligible women (*n *= 98), we defined a population-based cohort of 67,422 women.

### Baseline and five-year follow-up survey

Surveys of the cohort were conducted twice by self-administered questionnaire, the baseline in 1990 to 1994 and the five-year follow-up in 1995 to 1998. Of 67,422 women, a total of 55,886 women (83%) returned the baseline questionnaire. Of these, 1,510 who reported a history of cancer in the baseline survey were excluded, leaving 54,376 women as the baseline questionnaire respondents. Of the 67,422 women, we identified 62,788 as eligible for the five-year follow-up survey after exclusion of those who had died, moved out of the study area, or been lost to follow-up before the survey, of whom 52,485 (84%) returned the questionnaire. We also excluded 1,860 women who had a history of cancer at the five-year follow-up survey and 5,813 women who did not respond to the baseline questionnaire, leaving 44,812 women as the five-year follow-up questionnaire respondents.

### Exposure measurements

Information on beverages, including green tea, oolong tea, black tea, and coffee, was obtained in the baseline questionnaire in terms of frequency and amount using six precoded categories: less than one cup per week, one to two cups per week, three to four cups per week, and almost daily (further divided into one to two cups per day, three to four cups per day, and five or more cups per day). In the five-year follow-up questionnaire, the consumption of beverages, including two items of green tea, namely *Sencha *(first or second flush of green tea; that is, the first seasonal picking) and *Bancha *(third or fourth flush of green tea; that is, the late seasonal picking)*/Genmaicha *(blend of *bancha *and roasted brown rice), oolong tea, black tea, coffee and canned coffee was assessed in terms of frequency and amount using nine precoded categories: less than one cup per week, one to two cups per week, three to four cups per week, five to six cups per week, one cup per day, two to three cups per day, four to six cups per day, seven to nine cups per day, and ten or more cups per day. *Sencha *and *Bancha *are the two main types of green tea consumed in Japan, and are usually prepared by steeping the tea leaves in hot water. The amounts of *Sencha *or *Bancha/Genmaicha *consumed (ml per day) were computed by multiplying the frequency by the portion size for each beverage (120 ml per cup). Total green tea intake was defined as the sum of *Sencha *and *Bancha/Genmaicha *intake. The validity of green tea intake reported by the cohort was assessed using dietary records for 28 days (seven-day dietary records in four seasons) or 14 days. Spearman's correlation coefficients for green tea intake between the dietary record data and baseline questionnaire were 0.63 for Cohort I [20] and 0.43 for Cohort II (unpublished data).

### Follow-up

All registered women were followed from the start of the study period to 31 December 2006. Data on residential relocation were obtained from residential registries. Among the baseline respondents (*n *= 54,376), 2,962 women (5.4%) moved out of the study area and 201(0.4%) were lost to follow-up. The occurrence of cancer was identified by active patient notification from major local hospitals in the study area and data linkage with population-based cancer registries, with permission from the local governments responsible for the registries. Death certificates were used to supplement information on cancer incidence. Site of origin and histologic type were coded by members of our Study Group using the International Classification of Diseases for Oncology, Third Edition (ICD-O-3), code C500-509. Information on estrogen receptor (ER) and progesterone receptor (PR) status was collected from medical records or pathology reports. Up to the end of the study period, 586 new breast cancer cases were identified among the baseline questionnaire respondents (*n *= 54,376) and 362 cases among the five-year follow-up questionnaire respondents (*n *= 44,812). Diagnosis was microscopically verified in 95% of 586 cases, and based on death certificates only in 1.0%. Information on ER and PR status was available for 278 (47%) and 262 (45%) of 586 cases, respectively.

### Statistical analysis

Since the assessment of green tea intake differed between the baseline and five-year follow-up questionnaires, these were analyzed separately. We excluded 583 women with incomplete information for green tea from 54,376 women, leaving a total of 53,793 women, including 581 breast cancer cases, for inclusion in the baseline data analyses. We also excluded 1,173 women with incomplete information for *Sencha *or *Bancha/Genmaicha *from 44,812 women, leaving a total of 43,639 women, including 350 breast cancer cases, for inclusion in the five-year follow-up data analyses.

In the baseline data analyses, person-years of follow-up were calculated from the baseline survey (1990 to 1994) until the date of diagnosis of breast cancer, date of relocation from the study area, date of death, or end of the study period (31 December 2006), whichever occurred first. Similarly, person-years of follow-up were calculated from the five-year follow-up survey (1995 to 1998) until the date of diagnosis of breast cancer, date of relocation from the study area, date of death, or end of the study period (31 December 2006), whichever occurred first for the five-year follow-up data analyses.

The Cox proportional hazards model was used to estimate hazard ratio (HR) and 95% confidence intervals (CI) of breast cancer by green tea intake using the SAS program (PROC PHREG) (SAS Institute Inc., Cary, NC, USA). The following variables were used for adjustment as potential confounders: age, study area, age at menarche, menopausal status at baseline and age at menopause, number of births, age at first birth, height, body mass index, alcohol intake, smoking status, physical activity, exogenous hormone use, family history of breast cancer, oolong tea intake, black tea intake, coffee intake and canned coffee intake. Linear trends for HRs were tested in the Cox proportional hazards models using the exposure categories as ordinal variables. All *P-*values reported are two-sided, and significance level was set at *P *< 0.05.

## Results

During 733,667 person-years of follow-up (average follow-up, 13.6 years) for 53,793 women between 1990 to 1994 and 2006, a total of 581 cases of breast cancer were newly diagnosed and included in the baseline data analyses; while during 412,801 person-years of follow-up (average follow-up, 9.5 years) for 43,639 women between 1995 to 1998 and 2006, a total of 350 cases of breast cancer were newly diagnosed and included in the five-year follow-up data analyses.

Baseline characteristics of the study participants according to green tea intake are shown in Table 1. Approximately 12% of women drank green tea less than one cup per week while 27% drank five or more cups per day in the baseline data; and 22% and 30% of women did not drink *Sencha *and *Bancha/Genmaicha*, while 5.2% and 2.5% drank 10 or more cups per day, respectively, in the five-year follow-up data. Women who drank five or more cups per day in the baseline data tended to be older, live in towns or villages, be taller, have an earlier onset of first menstruation and fewer births, have a lower use of exogenous female hormones, drink less oolong tea and coffee, and consume more fruits and isoflavones. Similar characteristics was observed for women who drank 10 or more cups of *Sencha *per day in the five-year follow-up data, while women who drank 10 or more cups of *Bancha/Genmaicha *per day tended to be older, have later onset of first menstruation, earlier age at first birth and a larger number of births.

**Table 1:** Baseline characteristics according to green tea intake*

	Green tea assessed by baseline questionnaire			Green tea assessed by five-year follow-up questionnaire, *Sencha*			Green tea assessed by five-year follow-up questionnaire, *Bancha/Genmaicha*		
			
	Less than one cup per week	One to two cups per day	Five or more cups per day	Less than one cup per week	Two to three cups per day	Ten or more cups per day	Less than one cup per week	Two to three cups per day	Ten or more cups per day
No. of subjects	6,202	11,322	14,308	9,638	9,052	2,281	13,053	8,148	1,099
Age (year), mean	50.4	50.8	53.9	58.2	57.0	58.9	57.5	57.8	59.7
Residential area (town or village), %	40.9	57.3	68.5	58.7	66.4	73.2	66.9	64.0	60.2
Family history of breast cancer, %	0.7	1.2	1.1	0.9	1.5	0.8	1.3	1.1	0.9
Premenopausal women, %	46	47	31	21	24	15	23	22	14
Age at menopause (year), mean†	49.2	49.3	49.4	-	-	-	-	-	-
Age at menopause (50 to 54 years old), %	-	-	-	46	50	47	47	49	46
Age at menarche (year), mean†	15.0	14.6	14.7	15.0	14.6	14.7	14.7	14.7	14.9
Number of births, mean†	2.9	2.7	2.7	3.0	2.6	2.7	2.7	2.7	2.9
Age at first birth (year), mean†	24.8	25.0	24.9	24.6	25.0	25.0	25.0	24.8	24.6
Use of exogenous female hormones (current use), %	1.4	1.0	1.0	2.9	2.4	2.1	2.4	2.3	2.3
Height (cm), mean†	150.9	152.0	152.2	151.1	152.0	152.3	151.8	151.7	151.9
Body mass index (kg/m2), mean†	23.6	23.3	23.4	23.7	23.3	23.4	23.4	23.4	23.6
Smoking (current smoker), %	7.9	6.5	7.0	5.6	4.4	8.0	6.1	4.1	8.4
Alcohol drinking (regular drinker), %	11	14	11	9.7	13	13	13	11	12
Leisure-time physical activity (≥ once per week), %	15	18	18	-	-	-	-	-	-
Physical activity (metabolic equivalent to hours per day), mean†	-	-	-	32.0	31.9	32.2	31.8	32.1	32.4
Vitamin supplement user, %	20	19	20	12	16	15	15	15	13
*Bancha/Genmaicha *intake (≥ four cups per day), %	-	-	-	30	6.2	25	-	-	-
*Sencha *intake (≥ four cups per day), %	-	-	-	-	-	-	43	14	44
Oolong tea intake (≥ one cup per day), %	20	17	10	11	12	8.8	11	13	11
Black tea intake (≥ one cup per day), %	2.8	5.0	3.2	2.6	5.2	3.8	3.3	5.0	4.4
Coffee intake (≥ one cup per day), %	41	49	28	36	40	22	34	40	27
Canned coffee intake (≥ one cup per day), %	-	-	-	4.3	2.7	2.2	2.8	3.2	2.8
Total energy intake (kcal/day), mean†‡	1,770.0	1,764.0	1,805.2	1,770.1	1,923.5	2,194.0	1,833.9	1,934.6	2,185.8
Fish and shellfish intake (g/day), mean†‡	105.5	102.8	105.4	79.6	97.5	114.3	88.2	97.0	105.3
Meats intake (g/day), mean†‡	67.2	65.2	66.1	59.7	60.4	67.7	56.6	61.6	71.6
Vegetable intake (g/day), mean†‡	293.8	293.7	295.7	217.0	247.7	319.9	234.9	245.8	327.0
Fruit intake (g/day), mean†‡	175.9	177.3	180.8	208.5	269.9	359.3	243.1	270.1	323.7
Isoflavone intake (mg/day), mean†‡	31.6	32.2	33.5	37.5	43.3	54.7	41.5	42.0	54.8

* Three categories were chosen from six or nine precoded categories, respectively: less than one cup per week, one to two cups per week, three to four cups per week, one to two cups per day, three to four cups per day, and five or more cups per day for baseline questionnaire, and less than one cup per week, one to two cups per week, three to four cups per week, five to six cups per week, one cup per day, two to three cups per day, four to six cups per day, seven to nine cups per day, and ten or more cups per day for five to year follow-up questionnaire.

†Adjusted for age.

‡ Intake for each subject was estimated from the food frequency questionnaires based on a regression function derived from the validation study data (baseline questionnaire only).

We found no inverse association between green tea intake and the risk of breast cancer regardless of green tea intake as assessed by the baseline or five-year follow-up questionnaire (Table 2). Compared with women who drank less than one cup of green tea per week, the adjusted HR for women who drank five or more cups per day was 1.12 (95% CI 0.81 to 1.56; *P *for trend = 0.60) in the baseline data analyses. Similarly, compared with women who drank less than one cup of *Sencha *or *Bancha/Genmaicha *per week, adjusted HRs for women who drank 10 or more cups per day in the five-year data analyses were 1.02 (95% CI 0.55 to 1.89; *P *for trend = 0.48) for *Sencha *and 0.86 (0.34 to 2.17; *P *for trend = 0.66) for *Bancha/Genmaicha*. Moreover, compared with women who drank neither *Sencha *nor *Bancha/Genmaicha*, the adjusted HR for women who drank more than 1,320 ml per day was 1.29 (0.60 to 2.79; *P *for trend = 0.70). No substantial change was seen after further adjustment for other potential confounders such as residential area or dietary intake of meat, fish, vegetables, fruit, energy and isoflavones (data not shown). Further, no substantial change was seen in either baseline or five-year follow-up data after the exclusion of women within the first five years of follow-up to minimize the influence of existing preclinical conditions (data not shown), or after the exclusion of women who drank more than one cup of oolong tea or black tea per week to prevent the inclusion of other tea drinkers into the green tea intake reference category (data not shown).

**Table 2:** Hazard ratio and 95% confidence interval of breast cancer according to green tea intake

	Green tea intake assessed by baseline questionnaire																			
					
	Less than one cup per week	One to two cups per week			Three to four cups per week			One to two cup per day			Three to four cups per day			Five or more cups per day						*P* for trend
No. of cases	68	50			36			117			160			150						
Person-years	87,841	56,200			40,912			152,896			201,005			194,813						
Age- and area-adjusted HR (95% CI)	1.00	1.18	(0.82 to	1.71)	1.17	(0.78 to	1.75)	1.11	(0.82 to	1.50)	1.18	(0.88 to	1.59)	1.12	(0.83 to	1.51)				0.58
Multivariate HR (95% CI)*	1.00	1.19	(0.80 to	1.76)	1.13	(0.72 to	1.75)	1.13	(0.81 to	1.58)	1.17	(0.85 to	1.62)	1.12	(0.81 to	1.56)				0.60
	**Green tea intake assessed by five-year follow-up questionnaire**																			
		
	**Less than one cup per week**	**One to six cups per week**			**One cup per day**			**Two to three cups per day**			**Four to six cups per day**			**Seven to nine cups per day**			**Ten or more cups per day**			P for trend
*Sencha*																				
No. of cases	82	56			35			63			78			18			18			
Person-years	91,228	75,237			38,328			85,491			73,160			27,641			21,715			
Age- and area-adjusted HR (95% CI)	1.00	0.79	(0.56 to	1.12)	1.02	(0.68 to	1.52)	0.83	(0.59 to	1.16)	1.22	(0.88 to	1.69)	0.74	(0.44 to	1.26)	0.92	(0.55 to	1.55)	0.77
Multivariate HR (95% CI)†	1.00	0.71	(0.46 to	1.08)	0.97	(0.59 to	1.58)	0.85	(0.57 to	1.27)	1.25	(0.84 to	1.86)	0.68	(0.36 to	1.31)	1.02	(0.55 to	1.89)	0.48
*Bancha/Genmaicha*																				
No. of cases	107	90			38			69			30			7			9			
Person-years	123,189	99,201			42,254			76,532			46,563			14,733			10,329			
Age- and area-adjusted HR (95% CI)	1.00	1.02	(0.77 to	1.36)	1.05	(0.72 to	1.53)	1.09	(0.80 to	1.49)	0.80	(0.53 to	1.21)	0.58	(0.27 to	1.26)	1.02	(0.52 to	2.02)	0.41
Multivariate HR (95% CI)†	1.00	1.12	(0.80 to	1.58)	1.23	(0.79 to	1.92)	1.23	(0.85 to	1.78)	0.83	(0.51 to	1.35)	0.70	(0.28 to	1.76)	0.86	(0.34 to	2.17)	0.66
																				
	**Total green tea intake assessed by five-year follow-up questionnaire‡**																			
		
	**0**	**1 to 119 ml per day**			**120 to 299 ml per day**			**300 to 599 ml per day**			**600 to 959 ml per day**			**960 to 1319 ml per day**			**1320 ml + per day**			***P* for trend**
No. of cases	13	44			48			85			105			28			27			
Person-years	18,312	50,715			58,499			91,109			114,255			44,745			35,166			
Age- and area-adjusted HR (95% CI)	1.00	1.25	(0.67 to	2.32)	1.25	(0.67 to	2.32)	1.47	(0.81 to	2.67)	1.48	(0.82 to	2.67)	1.02	(0.52 to	2.00)	1.20	(0.61 to	2.36)	0.88
Multivariate HR (95% CI)§	1.00	1.08	(0.52 to	2.23)	1.26	(0.62 to	2.56)	1.59	(0.81 to	3.14)	1.43	(0.73 to	2.83)	0.89	(0.40 to	1.97)	1.29	(0.60 to	2.79)	0.70

*,†,§ Adjusted for age (continuous), area (10 public health centers), age at menarche (continuous), menopausal status at baseline (premenopausal women, age at menopause for postmenopausal women (* - 47, 48 to 50, 51 to 53, 54+; †,§ - 44, 45 to 49, 50 to 54, 55+)), number of births (0, 1, 2, 3, 4, 5+), age at first birth (-21, 22 to 25, 26 to 29, 30+, nulliparous), height (continuous), BMI (continuous), alcohol intake (non-drinkers, occasional drinkers, -150 (g per week) and 150+ (g per week) among regular drinkers (ethanol)), smoking status (never, past, current), *leisure time physical activity (no, one to three days per month, more than one day per week), †,§ daily physical activity (metabolic equivalent-hours per day) (continuous), exogenous hormone use (never, past, current), family history of breast cancer (yes, no), oolong tea intake (*less than one cup per week, one to four cups per week, one or more cups per day; †,§ less than 1 cup per week, one to six cups per week, one or more cups per day), black tea intake (*less than one cup per week, one to four cups per week, one or more cups per day; †,§ less than one cup per week, one to six cups per week, one or more cups per day), coffee intake (*less than one cup per week, one to four cups per week, one to two cups per day, three or more cups per day; †,§ less than one cup per week, one to six cups per week, one or more cups per day), †,§ canned coffee intake (less than one cup per week, one to six cups per week, one or more cups per day), and †Sencha and Bancha/Genmaicha intake.

Models for Sencha or Bancha/Genmaicha intake did not include these variables, respectively.

‡ Total green tea intake was defined as the sum of Sencha and Bancha/Genmaicha intake (ml per day).

To assess the potential influence of changes in green tea intake during the follow-up period on our findings, we first categorized subjects into three consumption groups for each survey: non-drinkers (corresponding to 'less than one cup per week'), 1 to 719 ml per day, and more than 720 ml per day (corresponding to 'more than five cup per day' in the baseline questionnaire). We next categorized subjects into three groups based on the combination of green tea intake calculated from the both the baseline and five-year follow-up questionnaires: non-drinkers for both questionnaires, more than 720 ml per day for both questionnaires, and other combinations. Compared with women who did not drink green tea for both questionnaires, adjusted HR was 1.12 (95% CI 0.45 to 2.83) for women who drank more than 720 ml per day for both questionnaires.

For analysis of cases by hormone receptor status and among women grouped by menopausal status, we re-categorized women into four groups for the baseline data and three for the five-year follow-up data. In the baseline data analyses, we found no inverse association between green tea intake and the risk of breast cancer regardless of hormone receptor-defined subtype (Table 3). Stratified analyses according to baseline menopausal status showed no remarkable difference between strata. Similar results were obtained when we analyzed the five-year follow-up data (data not shown). Additional stratified analyses according to dietary intake observed no remarkable difference between subgroups defined by dietary isoflavone and folate intake for either the baseline or five-year follow-up data (data not shown).

**Table 3:** Hazard ratio and 95% confidence interval of breast cancer according to subgroup analyses

	Green tea intake assessed by baseline questionnaire							
		
	Less than one cup per day	One to two cups per day		Three to four cups per day		Five or more cups per day		*P* for trend
*All subjects*								
No. of cases	154	117		160		150		
Age- and area-adjusted HR (95% CI)	1.00	1.01	(0.79 to 1.29)	1.08	(0.86 to 1.36)	1.02	(0.81 to 1.30)	0.75
Multivariate HR (95% CI)*	1.00	1.04	(0.80 to 1.35)	1.08	(0.84 to 1.39)	1.03	(0.80 to 1.34)	0.76
*ER+PR+ breast cancer*								
No. of cases	29	25		32		32		
Age- and area-adjusted HR (95% CI)	1.00	1.25	(0.73 to 2.17)	1.18	(0.69 to 2.00)	1.05	(0.62 to 1.79)	0.93
Multivariate HR (95% CI)*	1.00	1.33	(0.75 to 2.38)	1.35	(0.77 to 2.36)	1.02	(0.57 to 1.83)	0.92
*ER- PR- breast cancer*								
No. of cases	23	16		20		16		
Age- and area-adjusted HR (95% CI)	1.00	1.10	(0.58 to 2.11)	1.09	(0.58 to 2.05)	0.82	(0.42 to 1.60)	0.61
Multivariate HR (95% CI)*	1.00	1.13	(0.55 to 2.32)	1.23	(0.63 to 2.41)	0.81	(0.39 to 1.69)	0.69
*Premenopausal women*								
No. of cases	81	59		71		51		
Age- and area-adjusted HR (95% CI)	1.00	1.01	(0.72 to 1.43)	1.13	(0.81 to 1.58)	0.95	(0.66 to 1.37)	0.99
Multivariate HR (95% CI)*	1.00	1.05	(0.73 to 1.49)	1.12	(0.79 to 1.58)	0.97	(0.66 to 1.41)	0.99
*Postmenopausal women*								
No. of cases	70	56		86		96		
Age- and area-adjusted HR (95% CI)	1.00	1.01	(0.71 to 1.45)	1.04	(0.75 to 1.45)	1.05	(0.76 to 1.45)	0.76
Multivariate HR (95% CI)*	1.00	1.01	(0.67 to 1.50)	1.02	(0.71 to 1.48)	1.08	(0.75 to 1.55)	0.67

* Adjusted for age (continuous), area (10 public health centers), age at menarche (continuous), menopausal status at baseline (premenopausal women, age at menopause for postmenopausal women (-47, 48 to 50, 51 to 53, 54+)), number of births (0, 1, 2, 3, 4, 5+), age at first birth (-21, 22 to 25, 26 to 29, 30+, nulliparous), height (continuous), BMI (continuous), alcohol intake (non-drinkers, occasional drinkers, -150 (g/week) and 150+ (g/week) among regular drinkers (ethanol)), smoking status (never, past, current), leisure time physical activity (no, one to three days per month, more than one day per week), exogenous hormone use (never, past, current), family history of breast cancer (yes, no), oolong tea intake (less than one cup per week, one to four cups per week, one or more cups per day), black tea intake (less than one cup per week, one to four cups per week, one or more cups per day) and coffee intake (less than one cup per week, one to four cups per week, one to two cups per day, three or more cups per day).

In additional analyses to investigate the associations of oolong tea, black tea, and coffee intake with breast cancer risk, we found no inverse associations with the baseline (Table 4) or five-year follow-up data (data not shown).

**Table 4:** Hazard ratio and 95% confidence interval of breast cancer according to oolong tea, black tea and coffee intake as assessed by baseline survey

	Oolong tea intake							
				
	Less than one cup per week	One to four cups per week		One or more cups per day				*P* for trend
No. of cases	336	153		76				
Person-years	454,964	148,821		93,630				
Age- and area-adjusted HR (95% CI)	1.00	1.37	(1.13 to 1.67)	1.09	(0.85 to 1.41)			0.08
Multivariate HR (95% CI)*	1.00	1.34	(1.09 to 1.66)	0.98	(0.74 to 1.30)			0.40
	**Black tea intake**							
				
	**Less than one cup per week**	**One to four cups per week**		**One or more cups per day**				***P* for trend**
								
No. of cases	441	111		24				
Person-years	557,038	140,312		24,776				
Age- and area-adjusted HR (95% CI)	1.00	1.00	(0.81 to 1.24)	1.29	(0.85 to 1.96)			0.45
Multivariate HR (95% CI)*	1.00	0.84	(0.67 to 1.07)	1.30	(0.84 to 2.02)			0.80
	**Coffee intake**							
		
	**Less than one cup per week**	**One to four cups per week**		**One to two cups per day**		**Three or more cups per day**		***P* for trend**
								
No. of cases	161	180		173		63		
Person-years	233,697	213,979		210,969		69,940		
Age- and area-adjusted HR (95% CI)	1.00	1.20	(0.97 to 1.49)	1.18	(0.94 to 1.48)	1.30	(0.95 to 1.77)	0.09
Multivariate HR (95% CI)*	1.00	1.15	(0.91 to 1.46)	1.12	(0.87 to 1.43)	1.22	(0.87 to 1.71)	0.26

* Adjusted for age (continuous), area (10 public health centers), age at menarche (continuous), menopausal status at baseline (premenopausal women, age at menopause for postmenopausal women (-47, 48 to 50, 51 to 53, 54+)), number of births (0, 1, 2, 3, 4, 5+), age at first birth (-21, 22 to 25, 26 to 29, 30+, nulliparous), height (continuous), BMI (continuous), alcohol intake (non-drinkers, occasional drinkers, -150 (g/week) and 150+ (g/week) among regular drinkers (ethanol)), smoking status (never, past, current), leisure time physical activity (no, one to three days per month, more than one day per week), exogenous hormone use (never, past, current), family history of breast cancer (yes, no), green tea intake (less than one cup per day, one to two cups per day, three to four cups per day, five or more cups per day), oolong tea intake (less than one cup per week, one to four cups per week, one or more cups per day), black tea intake (less than one cup per week, one to four cups per week, one or more cups per day) and coffee intake (less than one cup per week, one to four cups per week, one to two cups per day, three or more cups per day). Models for oolong tea, black tea, or coffee intake did not include these variables, respectively.

## Discussion

In this population-based prospective cohort study, we found no overall association between green tea intake and the risk of breast cancer among Japanese women regardless of menopausal status. Our findings are in general agreement with those of three prospective studies, including two Japanese cohort studies, which found no association between green tea intake and breast cancer risk [12-14]. One noteworthy strength of the present over previous studies is the our remarkably wide variation in green tea intake, from women who drank green tea less than one cup per week to those who drank 10 or more cups per day. This strength argues against the possibility that the observed absence of associations with breast cancer risk is attributable to insufficient variation in green tea intake. Our findings therefore suggest that green tea intake within a usual drinking habit is unlikely to reduce the risk of breast cancer.

The other major strength of the present study was its prospective design, in which information was collected before the subsequent diagnosis of breast cancer, thereby avoiding the exposure recall bias inherent to case-control studies. Subjects were selected from the general population, the sample was large, the response rate to the questionnaire (more than 80%) was acceptable for study settings such as this, and the loss to follow-up (0.4%) was negligible. Furthermore, the cancer registry in the study population was of sufficient quality to reduce the possibility of misclassification of the outcome.

Several limitations of this study warrant mention. First, since green tea intake was assessed by self-administered questionnaire, misclassification may have been unavoidable, albeit that our validation study showed relatively high validity [20]. Changes in green tea intake during the follow-up period may also have caused misclassification. To assess the potential influence of this misclassification, we calculated HRs for women who drank more than 720 ml per day in both the baseline and five-year follow-up questionnaires versus those who did not drink green tea in either questionnaire. Although we found no association, we cannot deny the possibility that changes in green tea intake during the follow-up period influenced the findings, particularly considering that the questionnaires used in the baseline and five-year follow-up surveys were different. These misclassifications due to inaccurate measurement would in turn have attenuated the true association, which might be one reason for our results. However, we previously found an inverse association between green tea intake and the risk of distal gastric cancer among women [21], and between green tea intake and the risk of advance prostate cancer among men in the JPHC study using the same analytic approach, namely that exposure status was not updated during follow-up [22]. These findings would also argue against the possibility that the observed absence of associations with breast cancer risk was attributable to inaccurate measurement of green tea intake.

Second, in spite of a reasonably large cohort population (53,793 women) and long follow-up period (average 13.6 year), the number of breast cancer cases was relatively low (*n *= 581) in the baseline data analyses, reflecting the low incidence rate in Japan (age-standardized rate per 100,000 world population in 2002, 32.7 in Japan and 101.1 in United States for comparison) [23]. Although we found no association between green tea intake and breast cancer risk (HR for five or more cups per day versus less than one cup per week = 1.12 in the baseline data analyses), the 95% CI was 0.81 to 1.56, which was a relatively wide. Our relatively small sample size therefore cannot deny the possibility of a smaller increase or decrease in risk.

Third, although we measured and adjusted for several potential confounders in the statistical model as far as possible, the effects of confounding by unmeasured variables and residual confounding cannot be totally discarded.

Fourth, information on hormone receptor status was available for 45 to 47% cases in the present study. The major reason for the unknown cases is that collection of this information began in 2002, while data for 1990 to 2002 were obtained by retrospective review of medical records or pathology reports. The relatively small number of cases weakened the statistical power to detect the association. In addition, despite the generally high agreement between the enzyme-linked immunoassay and immunohistochemical techniques, differences in classification as well as interlaboratory variation may result in misclassification, which, if present, may have also attenuated the true association. These methodological limitations might partly explain our findings, which showed no inverse association between green tea intake and the risk of breast cancer regardless of hormone receptor-defined subtype. Thus, further research to clarify the differential associations by hormone receptor-defined subtype is warranted.

Our findings contradict those of three case-control studies, which showed an inverse association [9-11]. Given that all three previous prospective studies showed no association [12-14], these findings might have been influenced by recall and selection bias stemming from the case-control design. Meanwhile, for the three case-control studies conducted outside Japan [9-11], inconsistent findings might be in part explained by differences in the type of green tea and drinking method. In general, green tea contains catechins, minerals, and vitamins, and their contents vary among green tea types: *Sencha*, for example, one of most popular green teas in Japan, contains higher levels of tannin, vitamin C and folate than *Bancha/Genmaicha *[24]. The present study, however, found no overall association between green tea intake and the risk of breast cancer regardless of type. Moreover, levels of tea polyphenol and other nutrients in green tea varies according to preparation, amount of green tea leaves, frequency of renewing a tea batch in the pot, water temperature, brewing time, and so on. Although two Chinese case-control studies took account of several of these conditions, including green tea leaf amount, brew strength, and tea batch renewal frequency [9,10], no study has directly measured prediagnostic biomarkers of tea polyphenols. In this regard, we conducted a nested cased-control study within the JPHC study and found no overall association between plasma tea polyphenols and the risk of breast cancer [25]. Differences in tea polyphenols level due to green tea type and drinking method are therefore unlikely to explain these inconsistent findings, in any major way at least.

Alternatively, the inconsistencies reported in previous studies might be explained by possible effect modification by dietary factors and genetic polymorphisms [11,14,26]. In a case-control study in Asian-Americans, a risk-reducing effect of green tea was primarily observed among subjects whose soy intake was low [11], while a nested case-control study in Singapore showed a protective effect of green tea against breast cancer among women with high-activity genotypes of the *methylenetetrahydrofolate reductase *(*MTHFR*) and *thymidylate synthase *(*TYMS*) genes [14]. This association was stronger among those whose dietary folate intake was low. Subsequent studies, including our present study, however, have failed to replicate effect modification by soy and folate intake [9,13]. On the other hand, given a lack of information on genetic polymorphisms, the possibility cannot be excluded that green tea drinking is protective among subgroups of women.

Moreover, a recent cohort study in Shanghai showed a time-dependent interaction between green tea consumption and age of breast cancer onset: HRs for women who started green tea drinking at 25 years of age or younger versus non-green tea drinkers were 0.69 for premenopausal breast cancer and 1.61 for postmenopausal breast cancer [16]. This complex observation might also provide an explanation for the inconsistencies reported in previous studies. However, the present study was not able to examine time-dependent interactions because of a lack of information on green tea drinking habits, such as age at the beginning of green tea drinking and years of drinking.

Although oolong tea, black tea, and coffee are less popular than green tea in Japan, they are also rich in phenolic compounds and have been hypothesized to have a protective effect against the development of breast cancer: oolong tea and black tea contain higher levels of theaflavins and thearubigins than catechins, and coffee contains a high level of chlorogenic acid [1-3]. We also found no association of oolong tea, black tea, coffee and canned coffee intake with breast cancer risk. In addition to the relatively small number of women who drank oolong tea, black tea, and coffee, the potential influence of green tea intake on our findings was not fully excluded, albeit that we adjusted for green tea intake. Our findings for black tea, however, are consistent with those of a previous meta-analysis [27]. In contrast, our findings for coffee intake contradict a recent meta-analysis, which suggested a small risk-reducing effect of coffee intake on breast cancer: for example, relative risk (95% CI) per increment of two cups per day was 0.98 (0.96 to 1.00) based on 18 studies [28]. In addition to this small risk reduction, the small variation in coffee intake in our participants might have contributed in part to our findings.

## Conclusions

In this population-based prospective cohort study, we found no overall association between green tea intake and the risk of breast cancer among Japanese women. Our findings suggest that drinking green tea as a beverage is unlikely to reduce the risk of breast cancer regardless of green tea type and number of cups within a usual drinking habit.

## Abbreviations

CI: confidence interval; EGCG: (-)-epigallocatechin-3-gallate; ER: estrogen receptor; HR: hazard ratio; JPHC Study: Japan Public Health Center-based Prospective Study; PHC: public health centers; PR: progesterone receptor.

## Competing interests

The authors declare that they have no competing interests.

## Authors' contributions

MoI, MaI, SS, NS, TY, TS and ST were involved with the study concept and design. MoI, MaI, SS, NS, TY, TS and ST participated in the acquisition of data. MoI, MaI, SS, NS, TY, TS, WW and ST contributed to the analyses and interpretation of data. MoI conducted the statistical analyses and wrote the manuscript. All authors participated in the interpretation of results and critical revision of the manuscript for important intellectual content. All authors read and approved the final manuscript.
